# Exploratory genome-wide association analysis of response to ketamine and a polygenic analysis of response to scopolamine in depression

**DOI:** 10.1038/s41398-018-0311-7

**Published:** 2018-12-14

**Authors:** Wei Guo, Rodrigo Machado-Vieira, Sanjay Mathew, James W. Murrough, Dennis S. Charney, Matthew Grunebaum, Maria A. Oquendo, Bashkim Kadriu, Nirmala Akula, Ioline Henter, Peixiong Yuan, Kathleen Merikangas, Wayne Drevets, Maura Furey, J. John Mann, Francis J. McMahon, Carlos A. Zarate Jr., Yin Yao Shugart

**Affiliations:** 1 Statistical Genomics and Data Analysis Core, National Institute of Mental Health, National Institutes of Health, Bethesda, MD USA; 2 Department of Psychiatry and Behavioral Sciences, University of Texas Health Science Center at Houston, Houston, TX USA; 3 Menninger Department of Psychiatry and Behavioral Sciences, Baylor College of Medicine, Houston, TX USA; 4 Departments of Psychiatry and Neuroscience, Icahn School of Medicine at Mount Sinai, New York, NY USA; 5 Columbia University Medical Center/New York State Psychiatric Institute, New York, NY USA; 6 Department of Psychiatry, Perelman School of Medicine, University of Pennsylvania, Philadelphia, PA USA; 7 Experimental Therapeutics and Pathophysiology Branch, National Institute of Mental Health, National Institutes of Health, Bethesda, MD USA; 8 Human Genetics Branch, National Institute of Mental Health, National Institutes of Health, Bethesda, MD USA; 9 Section on PET Neuroimaging Sciences, National Institute of Mental Health, National Institutes of Health, Bethesda, MD USA; 10 Genetic Epidemiology Branch, National Institute of Mental Health, National Institutes of Health, Bethesda, MD USA; 11 Janssen Pharmaceuticals, Neuroscience Research and Development, La Jolla, CA USA; 12 Departments of Psychiatry and Radiology, College of Physicians and Surgeons, Columbia University, New York State Psychiatric Institute, New York, NY USA

**Keywords:** Depression, Predictive markers

## Abstract

Growing evidence suggests that the glutamatergic modulator ketamine has rapid antidepressant effects in treatment-resistant depressed subjects. The anticholinergic agent scopolamine has also shown promise as a rapid-acting antidepressant. This study applied genome-wide markers to investigate the role of genetic variants in predicting acute antidepressant response to both agents. The ketamine-treated sample included 157 unrelated European subjects with major depressive disorder (MDD) or bipolar disorder (BD). The scopolamine-treated sample comprised 37 unrelated European subjects diagnosed with either MDD or BD who had a current Major Depressive Episode (MDE), and had failed at least two adequate treatment trials for depression. Change in Montgomery–Asberg Depression Rating Scale (MADRS) or the 17-item Hamilton Depression Rating Scale (HAM-D) scale scores at day 1 (24 h post-treatment) was considered the primary outcome. Here, we conduct pilot genome-wide association study (GWAS) analyses to identify potential markers of ketamine response and dissociative side effects. Polygenic risk score analysis of SNPs ranked by the strength of their association with ketamine response was then calculated in order to assess whether common genetic markers from the ketamine study could predict response to scopolamine. Findings require replication in larger samples in light of low power of analyses of these small samples. Neverthless, these data provide a promising illustration of our future potential to identify genetic variants underlying rapid treatment response in mood disorders and may ultimately guide individual patient treatment selection in the future.

## Introduction

The development of rapid-acting treatments for individuals with major depressive disorder (MDD) or bipolar depression who fail to respond to conventional antidepressant treatments is an urgent public health priority, particularly because of the increased risk of suicide in patients with treatment-resistant depression^1–3^. Accumulating evidence indicates that a single infusion of the glutamatergic modulator ketamine can produce rapid, robust, and relatively sustained antidepressant effects within hours in patients with both non-treatment-resistant and treatment-resistant MDD and bipolar depression^4–11^. The anticholinergic agent scopolamine has also shown promise as a rapid-acting antidepressant^12,13^. Similar to ketamine, scopolamine’s mechanism of action is thought to stem from the convergent activation of synaptic plasticity and synaptogenesis^14^, with effects on glutamatergic activity occurring via antagonistic effects at muscarinic receptors^15^. Identifying the specific mechanisms and targets associated with antidepressant response to ketamine compared with scopolamine, as well as subgoups associated with treatment response, could facilitate personalized treatment selection in individuals with major depression.

The rapid onset of ketamine’s antidepressant effects offers a unique opportunity to examine potential biomarkers of response versus non-response within a short period of time^16^. Previous studies have explored biomarkers of antidepressant response to ketamine via several avenues, including proton magnetic resonance spectroscopy (MRS) measures of glutamate, glutamine, Glx (glutamate + glutamine), and/or gamma-aminobutyric acid (GABA) levels; structural and functional magnetic resonance imaging (MRI);^17^ positron emission tomography (PET) measures of metabotropic glutamatergic receptor (mGluR5) binding;^18^ magnetoencephalography (MEG) assessments of changes in synaptic plasticity;^19^ polysomnography;^20^ actigraphy;^21^ and biochemical measures^22–27^. While the identification of biomarkers associated with response to rapid-acting antidepressants is clear in the early stages of testing and development, such biomarkers could eventually offer promising additions or alternatives to the traditional rating scales used to assess clinical severity and outcome in depression, as well as suggest key avenues for personalized treatment.

In this context, elucidating the genetic variants that predict treatment response can potentially provide important biological information about the heterogeneity of depression (treatment-resistant and non-treatment-resistant), which may ultimately be relevant for clinical translation^28^. However, translation of these findings into clinical practice is limited by the small sample sizes and inconsistent findings^29^. More recently, two reports demonstrated the potential of pharmacogenetics in mood disorders. In the first study, 280 depressed subjects were randomized in a double-blind study comparing pharmacogenetic-guided treatment testing versus treatment as usual. Although no significant association was identified between genetic variation and sustained antidepressant response rate to a particular treatment, pharmacogenetic-guided treatment testing resulted in a higher response rate than treatment as usual at 12 weeks^30^. In the second study, an international collaborative effort identified a potential marker of lithium response using a genome-wide association study (GWAS) approach^31^.

The present study examined genetic markers of ketamine response and in a sample of patients with treatment-resistant depression, either MDD or BD in a current MDE. A second set of analyses was performed in an independent sample who were treated with scopolamine. Given the potential overlap between the targets and pathways underlying rapid antidepressant response to ketamine and scopolamine, we further explored this potential caveat using a polygenic risk score (PRS) approach^32,33^. PRS is a quantitative measure of the total contribution of common genetic variation to a trait and is calculated as a sum of multiple single-nucleotide polymorphisms (SNPs) alleles associated with an individual’s traits, typically weighted by effect sizes estimated from a GWAS study. PRS analysis has been successfully used to predict antidepressant efficacy in pharmacogenetic trials^34–36^. Furthermore, psychotomimetic symptoms, dissociation, and hemodynamic changes are well-known side effects of ketamine, and dissociative side effects are significantly correlated with antidepressant response^37^. Therefore, a separate GWAS was carried out on the dissociative side effects in a set of patients who were treated with ketamine.

## Materials and methods

### Sample

Subjects in the study comprised 326 patients (18–68 years old) diagnosed with MDD or BD, as assessed via DSM-IV-TR criteria^38^ with a current Major Depressive Episode (MDE). Patients enrolled in this study with ketamine were also required to have a history of treatment-resistance, defined as a current or past history of lack of response to at least two adequate antidepressant trials^39^. Patients were evaluated at four US research centers: the NIMH Intramural Program (*n* = 240), the Icahn School of Medicine at Mount Sinai (*n* = 22), the Baylor College of Medicine (*n* = 35), and Columbia University (*n* = 29). Additional details about these samples and the studies from which they were drawn have been previously published^6,10,11,40,41^. Briefly, clinical studies were randomized, placebo-controlled, double-blind trials where subjects received a single IV ketamine infusion (0.5 mg/kg) over 40 min. All sites had institutional review board (IRB) approval, and written informed consent was provided by all participants before entry into their relevant study (www.clinicaltrials.gov, Trial registration number: NCT00024635). Subjects were not selected for treatment-resistance. In the NIMH center, 127 subjects received ketamine infusions with the same methods as those at the other three centers. In addition, 69 subjects received a single IV scopolamine infusion (4 µg/kg over 60 min). Details of the studies have previously been published^12,13^.

Across both the ketamine analysis and the ketamine vs. scopolamine analysis, the primary outcome measures were the Montgomery–Asberg Depression Rating Scale (MADRS) or the 17-item Hamilton Depression Rating Scale (HAM-D). In the ketamine analysis, the proportional change in MADRS scores at day 1 (24 h post-treatment) from baseline was considered as the primary depression score; proportional change in HAM-D score was used when MADRS scores were not available. In the scopolamine analysis, change in MADRS score at day 3 from baseline was considered the primary depression score, as this was the earliest time point obtained in the scopolamine studies and closest to the day 1 assessment used in the ketamine analysis. At the baseline assessment on day 3, subjects had received only one scopolamine infusion. The study of the dissociative side effects was carried on with the Clinician-Administered Dissociative States Scale (CADSS), and the difference between CADSS scores at 40 min from baseline was used as the phenotype in the association studies of dissociation effects.

### GWAS analyses

Approximately 900,000 SNPs were genotyped on genotyped on Infinium OmniExpress and Infinium OmniExpressExome chips (Illumina Inc, San Diego, CA, USA) for 326 individuals in three phases. Prior to the imputation analysis, the restrictive quality control was executed within each phase and merged genotype data using PLINK v1.09. In summary, one duplicated subject was excluded; four subjects were excluded because they had a call rate < 0.98, no subject was excluded due to an absolute value of F inbreeding coefficient estimate F_HET > 0.20, and three samples were excluded due to unambiguous genotypic sex. Genetic relatedness between samples was examined through pairwise identity by descent (IBD) estimation; four samples were excluded when we retained only one member of each pair of samples with IBD coefficients > 0.2. SNPs were removed from the pre-imputation dataset if they had a call rate < 0.98, a minor allele frequency (MAF) < 0.01, or a *p*-value of Hardy–Weinberg equilibrium (HWE) < 1×10^−6^. Further, batch effects were checked by examining the pair-wised allele frequency difference in three genotyping phases, and SNPs were removed when *p*-value of Hardy–Weinberg equilibrium (HWE) < 0.001. After data cleaning, a total of 534,747 SNPs were included on 314 samples for imputation. Of these, 197 and 69 samples had nonmissing phenotypes in the ketamine and scopolamine studies, respectively.

Genotype imputation was conducted using the IMPUTE2 software program (version 2.1.2)^42,43^, using haplotypes from all 2504 individuals in the 1000 Genomes Phase 3 (October 2014 Data Release) as a reference panel. The haplotypes were phased using *SHAPEIT2* (version v2.r644), which can perform the pre-phasing step for the study genotypes to produce “best-guess” haplotypes. The imputed SNPs with low imputation quality were excluded if IMPUTE2 info was < 0.6, or IMPUTE2 certainty was < 0.8, or MAF was < 0.01.

Population structure was assessed using a multidimensional scaling (MDS) plot as shown in Figures S1; European samples were selected based on the MDS plot of the ketamine/scopolamine samples and four HapMap samples (https://www.genome.gov/10001688/international-hapmap-project/). After data were selected using dimensional data reduction techniques to remove samples falling outside the European genetic cluster, 157 and 37 subjects of European ancestry remained for the ketamine and scopolamine analyses, respectively. Only European samples were used in the statistical analysis in this study, and the sample summary was shown in Table 1. Among the 157 ketamine samples, 76 were male and 81 were female (18–68 years old; mean = 44.5, SD = 12.4). Among the 37 scopolamine samples, 18 were male and 19 were female (18–55 years old; mean = 35.1, SD = 10.4). Furthermore, 90 European samples were used in the GWAS of dissociation effects with nonmissing CADSS scores in the NIMH samples treated with ketamine. Among these 90 samples, 46 were male and 44 were female (20–65 years old; mean = 43.4, SD = 12.4).

**Table 1:** Summary of statistical analyses

Study	Infusion	Phenotype	Number of samples	Number of males	Number of females	Age range	Mean of age	SD of age
Antidepressant effects to ketamine	Ketamine	Depression Score (proportional change of MADRS or HAM-D at day 1)	157	76	81	18–68	44.54	12.37
Dissociation effects	Ketamine	change of CADSS_at 40 min	90	46	44	20–65	43.44	12.41
Antidepressant effects to scopolamine	Scopolamine	proportional change of MADRS at day 3	37	18	19	18–55	35.08	10.37

The GWAS analyses were conducted to assess antidepressant effects to ketamine and scopolamine separately, and no participants overlapped across the two studies^34–36^. The GWAS study of the dissociative side effects was also carried on. Association tests were conducted using the imputation dosage files using the PLINK software. Within the GWAS results, about 6,000,000 SNPs remained after excluding SNPs where the INFO was < 0.6 or INFO > 1.2, where INFO was defined as the *R*^2^ quality metric or information content in PLINK output.

### Polygenic risk score (PRS) analysis

To explore the genetic relationship between antidepressant response to ketamine and scopolamine, we utilized PRSice to conduct a standard PRS analysis^44^. The details of the PRS analysis are as follows. We obtain GWAS summary statistics (*p*-values and β’s) in the discovery sample (Ketamine sample), then obtain independent target samples with genome-wide data (Scopolamine). Following that, we use the overlapped SNPs between discovery and target samples with *p* *<* 0.5 in the GWAS study of the discovery sample before dealing with association redundancy due to linkage disequilibrium (LD). The SNPs defined in step 3 were pruned based on *r*^2^ < 0.5 or *r*^2^ < 0.2, where *r*^2^ was a measure of LD that typically based on comparisons of the observed frequencies of haplotypes to the frequencies expected. We restrict to SNPs based on predetermined significance thresholds. SNPs with *p* *<* 0.01, 0.05, 0.1 0.2, 0.3, 0.4, and 0.5, were considered in this PRS analysis.Within each pruned SNP set under each significance threshold, a quantitative aggregate risk score (PRS) was calculated for each individual in the target sample, defined as a sum across SNPs of the number of reference alleles (0, 1, or 2) at that SNP multiplied by the effect size measures (β’s) for that SNP estimated from the discovery sample. Association of aggregate risk score (PRS) and actual depression score (defined as MADRS or HAM-D17 score at Day 3 in the scopolamine sample) was performed with linear regression adjusted for gender, age, and 10 principal components to control for population stratification of the target sample. The R^2^ value, as a goodness-of-fit measure for linear regression models, was calculated to estimate the proportion of variance explained by the aggregate risk score.

## Results

### GWAS study

Association *p*-values in the GWAS analyses are reported in quantile–quantile plots (Figure S2) and Manhattan plots (Figure S3). Quantile–quantile plots compare observed versus expected test statistics distributions. The genomic control inflation factor, λ, was 1.008 for the ketamine GWAS studies, suggesting no evidence of residual population stratification or systematic technical artifacts. The *p*-values for all imputed SNPs are provided in the Manhattan plots (Figure S3).

No SNP exceeded the genome-wide threshold for significance of 5 × 10^−8^. The 31 SNPs with *p*-values < 1×10^−5^ from the ketamine GWAS study are shown in Table S1. Eight LD-independent loci were observed with *p*-values < 1 × 10^−5^ (Table 2). The associated SNPs were annotated using SNPsnap software^45^. The top-ranked SNP was rs55945116 (*p* = 5.93 × 10^−7^; BETA = 23.33), which lies wholly within SEC11A. SEC11A was SEC11 homolog A, signal peptidase complex subunit, and it is a protein coding gene linked to cell migration and invasion, gastric cancer, and lymph node metastasis. It is important to note that the SNP rs55945116 had an eQTL with *p*-value of 2.5×10^−8^ in the Nerve-Tibial tissue in the GTEx Analysis (https://www.gtexportal.org/home/, dbGaP Accession phs000424.v7.p2). The SEC11A, CRIM1, MAB21L3, SLC22A15, and C18orf42 genes contain eQTLs in the GTEx brain tissues including cortex, cerebellar hemisphere, caudate, and anterior cingulatecortex with *p*-values < 1 × 10^−5^. The number of brain samples in the V7 release ranges from 80 to 144 for different brain tissues, limiting the statistical power to detect brain eQTLs. The SNP rs112647602 (*p* = 4.82 × 10^−6^; BETA = 23.14) was located on chromosome 6 in a non‐coding region close to pseudogene KRASP1 and FAM83B, that codes for a regulatory protein probably involved in phosphoinositide 3‐kinase/protein kinase B (PI3K/AKT) and mitogen‐activated protein kinase signaling^46^. Moreover, a SNP rs16885979, as a LD-friend SNP that located 29 kb away at rs112647602, was found to be associated with resting as an actigraphic daytime and night sleep phenotype (*p*-values = 8 × 10^−8^)^47^. The regional association plot of rs55945116 and rs112647602 was illustrated in Figures S4 and S5.

**Table 2:** Genomic regions with *p* < 1×10^−5^ in the genome-wide association study (GWAS) on the antidepressant effects to ketamine

CHR	SNP	BP	A1	A2	FRQ	INFO	BETA	SE	*P*	Gene (distance)	Protein (distance)
15	rs55945116	85220113	G	C	0.7892	0.9432	23.3347	4.4624	5.93E-07	SEC11A (0)	SEC11A (0)
6	rs112647602	54596755	G	A	0.7875	0.871	−23.135	4.8666	4.82E-06	KRASP1 (38626)	FAM83B (114814)
4	rs1846786	131919702	T	G	0.6951	0.8884	−19.0893	4.0582	5.96E-06		
22	rs5997786	31254036	C	T	0.7995	0.8801	22.0315	4.6925	6.19E-06	OSBP2 (0)	OSBP2 (0)
2	rs1524145	36122388	G	T	0.5	0.9682	−17.3121	3.6948	6.44E-06	MRPL50P1 (172180)	CRIM1 (460681)
1	rs6689906	116683540	C	T	0.9364	0.9278	35.6552	7.6306	6.80E-06		MAB21L3 (29164)
1	rs79749176	116461018	T	C	0.9468	0.9306	39.0894	8.4686	8.63E-06		SLC22A15 (58101)
18	rs75908125	5155552	G	C	0.864	1.0926	22.8451	4.9849	9.91E-06	C18orf42 (0)	C18orf42 (0)

*Chr* chromosome, *SNP* single-nucleotide polymorphism, *A1* reference allele, *A2* alternative allele, *FRQ* frequency of A1 allele in all samples, *INFO* information score of association, *P* *p*-values of GWAS, gene (distance) gene name and distance to the start site of nearest gene the SNP is located within, protein (distance) protein name and distance to nearest protein coding gene start site

### Dissociative side effects

No SNP exceeded the genome-wide threshold for significance of 5 × 10^−8^ in the dissociation study. The 52 SNPs with *p*-values < 1 × 10^−5^ from the ketamine GWAS study are shown in Table S2. Twelve LD-independent loci were observed with *p*-values < 1 × 10^−5^ (Table 3). Association *p*-values in the GWAS analyses the CADSS dissociative score are reported in quantile–quantile plots (Figure S6) and Manhattan plots (Figure S7). The genomic control inflation factor, λ, was 1.012 for the ketamine GWAS studies, suggesting no evidence of residual population stratification or systematic technical artifacts. The top-ranked SNP was rs17211233 (*p* = 1.9 × 10^−7^; BETA = −27.0), which lies wholly within ROBO2 (roundabout guidance receptor 2). The regional association plot of rs17211233 was illustrated in Figure S8. The ROBO2 gene was broadly expressed in the adult brain with the highest expression in the ventral midbrain, hippocampus, and cerebellum^48^. And ROBO2 determines subtype-specific axonal projections of trigeminal sensory neurons^49^. The ROBO2, SPRED2, AMOTL1, C20orf196, and FAM179A genes contain eQTLs in the GTEx brain tissues including amygdala, cortex, cerebellar hemisphere, caudate, and anterior cingulatecortex with *p*-values < 1 × 10^−5^. Moreover, the SPRED2 gene was indentified as a critical regulator of synaptic transmission in different brain regions and as a new regulator of BDNF/TrkB pathways, and SPRED2 deficiency could result in OCD-like behavior^50^.

**Table 3:** Genomic regions with *p* < 1×10^−5^ in the genome-wide association study (GWAS) on the dissociation effects to ketamine

CHR	SNP	BP	A1	A2	FRQ	INFO	BETA	SE	*P*	Gene (distance)	Protein (distance)
5	rs17211233	80368763	T	C	0.9389	0.9349	−26.9757	4.7058	1.90E-07	RASGRF2 (0)	RASGRF2 (0)
3	rs1400237	76172232	T	G	0.0778	1.0706	21.4345	4.165	2.02E-06	ROBO2 (0)	ROBO2 (0)
9	rs12236015	122817820	C	T	0.833	1.1168	−13.9838	2.7763	3.10E-06		
3	rs9713737	76297174	A	G	0.244	0.9868	13.5351	2.7481	4.79E-06	ROBO2 (0)	ROBO2 (0)
2	rs77987715	65955079	A	G	0.6547	1.0199	−11.7152	2.3832	4.97E-06		SPRED2 (295308)
4	rs3900502	189869233	C	A	0.4877	0.8444	−12.2832	2.5129	5.53E-06		TRIML1 (808660)
11	rs3018154	94420308	C	T	0.9111	1.0392	−19.9461	4.0971	5.97E-06		AMOTL1 (19289)
20	rs1287071	5828375	A	G	0.7111	1.1346	−11.3492	2.3349	6.15E-06	C20orf196 (0)	C20orf196 (0)
2	rs11127199	29213561	G	A	0.6335	0.995	11.445	2.3621	6.53E-06	FAM179A (0)	FAM179A (0)
3	rs4855976	76267368	A	G	0.0702	0.7747	23.7477	4.9636	8.24E-06	ROBO2 (0)	ROBO2 (0)
20	rs11907319	42923299	T	C	0.8099	0.9753	14.3351	3.0037	8.63E-06	FITM2 (16510)	FITM2 (16510)
15	rs4558394	98322385	A	G	0.85	0.9108	−15.6495	3.2958	9.46E-06	LINC00923 (0)	LINC00923 (0)

*Chr* chromosome, *SNP* single-nucleotide polymorphism, *A1* reference allele, *A2* alternative allele, *FRQ* frequency of A1 allele in all samples, *INFO* information score of association, *P* *p*-values of GWAS, *gene (distance)* gene name and distance to the start site of nearest gene the SNP is located within, *protein (distance*) protein name and distance to nearest protein coding gene start site

### Polygenic risk score analysis

**Fig. 1:**
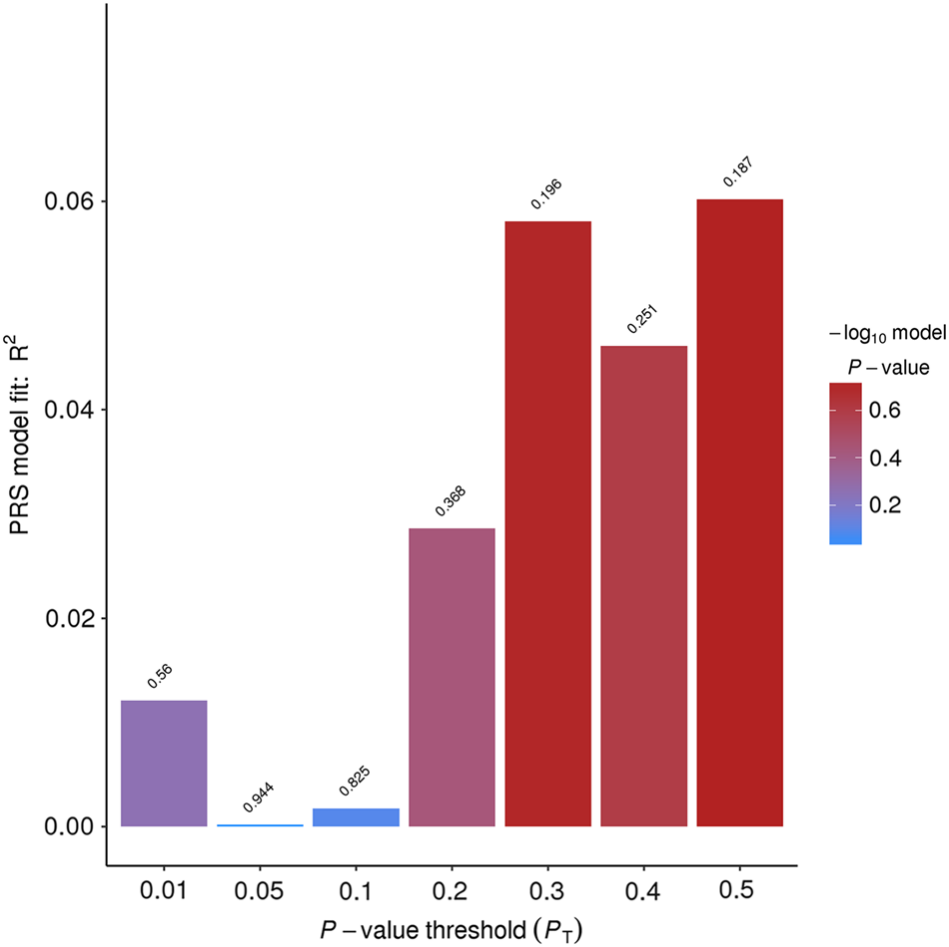
Polygenic risk score (PRS) analysis. The target sample comprised 37 subjects of European ancestry treated with scopolamine. The discovery sample comprised 157 subjects of European ancestry treated with ketamine. The variance explained in the target sample is based on risk scores derived from an aggregated sum of weighted single nucleotide polymorphism (SNP) risk allele effect sizes estimated from the discovery samples at seven significance thresholds (*p* *<* 0.01, 0.05, 0.1, 0.2, 0.3, 0.4, and 0.5). The *y*-axis indicates *R*^2^ value for the PRS fit model

SNP effect sizes derived from the individual ketamine GWAS analyses were used to calculate PRS and predict scopolamine phenotype score in individuals of European ancestry. As noted above, the number of SNPs in each group was selected based on predetermined GWAS significance thresholds (*p* *<* 0.01, 0.05, 0.1, 0.2, 0.3, 0.4, and 0.5, respectively (Fig. 1, Table S3). Overall, 97,129 SNPs with *p* < 0.5 in the ketamine study explained 6% of the variance in scopolaminet outcome (PRS *p*-value = 0.19). As a comparsion, the proportion of variance explained in the BD data by risk scores from the MDD data was previously estimated as about 0.5% in the pairwise cross-disorder polygene analysis^51^. Therefore, the antidepressant response-related variance of 6% suggests a substantial genetic overlap in antidepressant response to ketamine and scopolamine. As mentioned above, the number of scopolamine samples is 37, limiting the power of the PRS analysis, and final determination of this will require more data. To increase the sample size, the additional PRS anlaysis was also considered using all 69 scopolamine samples regardless of the confounding effects of population structure and results were shown in Table S3. Overall, SNPs with *p* < 0.5 in the ketamine study significantly explained 11% of the variance in 69 scopolaminet outcome (PRS *p*-value = 0.007). In addition, comparable results were obtained in the PRS analysis when using LD *r*^2^ < 0.5 to prune SNPs as shown in Table S3.

## Discussion

The present GWAS sought to identify potential markers of response to ketamine in subjects diagnosed with either MDD or BD; a second set of analyses was performed in an independent sample of MDD or BD subjects treated with scopolamine in order to investigate the potential role of genetic variants in predicting antidepressant response to both agents. We found that no SNP exceeded the genome-wide threshold for significance of 5 × 10^−8^. However, eight LD-independent loci had *p*-values < 1 × 10^−5^. The top-ranked SNP was rs55945116 (*p* = 6.0 × 10^−7^ with an effect size of 23.33), which is located within the SEC11A gene. The SEC11A, KRASP1, and FAM83B genes may help accelerate progress from genetic studies to the biological knowledge in antidepressant response to ketamine. The top-ranked SNP was rs17211233 (*p* = 1.9 × 10^−7^; BETA = −27.0) in the GWAS study on the dissociation effects to ketamine, which lies wholly within the RASGRF2 gene. In addition, ROBO2 and SPRED2 genes were indentified as promising factors in the dissociation effects to ketamine. It should be noted that the small sample sizes of this GWAS could inflate the type I error rate as well as reduce power for detecting truly associated genetic markers. Replication and extension of these findings are needed in studies with much larger samples.

Despite the exploratory nature of the finding, our study constitutes the first endeavor to use PRS to identify potential genetic overlap between rapid antidepressant response to ketamine and scopolamine. Specifically, we investigated a GWAS dataset for ketamine/scopolamine treatment response and applied a standard PRS approach to infer the variance explained by potentially associated SNPs. No SNP exceeded the genome-wide threshold for significance. However, the results of the polygenic analysis presented here suggested potential genetic overlap between rapid antidepressant response to ketamine and scopolamine. Interestingly, a highly polygenic model might suggest genetically influenced individual differences across brain function and development that may provide a diathesis model for depression, perhaps in the same way that a variety of growth and metabolic pathways may influence height in humans.

Findings revealed that genetic variants associated with ketamine response accounted for ~6% of the variance in scopolamine response, suggesting modest potential genetic overlap in predictors of response to these agents. Though intriguing, the shared genetic influence on response to ketamine and scopolamine did not reach statistical significance in the European samples, likely due to the underpowered analyses. Furthermore, genetic variants associated with ketamine response significantly explained 11% of the variance in 69 scopolaminet outcome (PRS *p*-value = 0.007). This finding needs to be illustrated with caution because of possible confounding effects caused by population stratification when both European and non-European samples are used. However, previous findings from clinical and genetic epidemiologic studies, and preclinical studies indicateds the possibility of genetically-based refinements in drug effect measures^52^. Recent preclinical studies have found that ketamine’s rapid antidepressant effects may stem from rapid increases of spine synapses in the prefrontal cortex, which presumably reverse the deficits caused by chronic stress^53^. This is believed to result in a rapid but transient burst of glutamate resulting from dishibition of glutamate transmission, followed by an increase in brain-derived neurotrophic factor (BDNF) release and activation of downstream signaling pathways that stimulate synapse formation. In parallel, recent studies have demonstrated that the rapid-acting antidepressant effects of scopolamine, a muscarinic receptor antagonist, are also associated with increased glutamate transmission and synapse formation^54^. Therefore, it is possible that despite differences in the immediate effects of the two antidepressants, ketamine, and scopolamine may have convergent outcomes in their downstream targets.

The preliminary nature of these results precludes our ability to translate the findings into prediction of clinical response or dissociative effects at the individual level. However, these findings provide an illustration of the future potential of this approach in guiding treatment of treatment-resistant depression and its side effects. Progress in elucidating the mechanisms underlying response to these rapid-acting antidepressants may facilitate our ability to apply the tools of molecular genetics to inform personalized treatment strategies for patients who suffer from treatment-resistant depression and bipolar.

## Electronic supplementary material

Supplemental Material

Figure S1

Figure S2

Figure S3

Figure S4

Figure S5

Figure S6

Figure S7

Figure S8
