# Correction: Exploratory genome-wide association analysis of response to ketamine and a polygenic analysis of response to scopolamine in depression

**DOI:** 10.1038/s41398-019-0442-5

**Published:** 2019-03-05

**Authors:** Wei Guo, Rodrigo Machado-Vieira, Sanjay Mathew, James W. Murrough, Dennis S. Charney, Matthew Grunebaum, Maria A. Oquendo, Bashkim Kadriu, Nirmala Akula, Ioline Henter, Peixiong Yuan, Kathleen Merikangas, Wayne Drevets, Maura Furey, J. John Mann, Francis J. McMahon, Carlos A. Zarate, Yin Yao Shugart

**Affiliations:** 1 Statistical Genomics and Data Analysis Core, National Institute of Mental Health, National Institutes of Health, Bethesda, MD USA; 2 Department of Psychiatry and Behavioral Sciences, University of Texas Health Science Center at Houston, Houston, TX USA; 3 Menninger Department of Psychiatry and Behavioral Sciences, Baylor College of Medicine, Houston, TX USA; 4 Departments of Psychiatry and Neuroscience, Icahn School of Medicine at Mount Sinai, New York, NY USA; 5 Columbia University Medical Center/New York State Psychiatric Institute, New York, NY USA; 6 Department of Psychiatry, Perelman School of Medicine, University of Pennsylvania, Philadelphia, PA USA; 7 Experimental Therapeutics and Pathophysiology Branch, National Institute of Mental Health, National Institutes of Health, Bethesda, MD USA; 8 Human Genetics Branch, National Institute of Mental Health, National Institutes of Health, Bethesda, MD USA; 9 Section on PET Neuroimaging Sciences, National Institute of Mental Health, National Institutes of Health, Bethesda, MD USA; 10 Genetic Epidemiology Branch, National Institute of Mental Health, National Institutes of Health, Bethesda, MD USA; 11 Janssen Pharmaceuticals, Neuroscience Research and Development, La Jolla, CA USA; 12 Departments of Psychiatry and Radiology, College of Physicians and Surgeons, Columbia University, New York State Psychiatric Institute, New York, NY USA

**Correction to: Translational Psychiatry**

10.1038/s41398-018-0311-7 Published online 14 December 2018

Michael F. Grunebaum’s name was misspelled/misstated as “Gruenbaum M.F.” in the original Article. This has now been updated in the HTML and PDF versions of this Article.

