# Supplementary material for: Exploratory genome-wide association analysis of response to ketamine and a polygenic analysis of response to scopolamine in depression
Source: Transl Psychiatry. 2018 Dec 14;8:280. doi: 10.1038/s41398-018-0311-7 (PMC6294748; doi:10.1038/s41398-018-0311-7)
Supplement: Supplementary file 1 — Supplemental Material [file 41398_2018_311_MOESM1_ESM.docx]

**Supplementary Figure Legends**

**Figure S1.** Population structures identified by multidimensional scaling of the ketamine data. The first two principal components are plotted on both 314 ketamine samples (in red) and four Hapmap samples. One hundred and sixty samples within the red circle were selected as being of European ancestry. For the Hapmap samples (used as a reference population), CEU indicates Utah residents with Northern and Western European ancestry from the Centre d'Etude du Polymorphisme Humain (CEPH) collection; CHD indicates Chinese in Metropolitan Denver, Colorado; MEX indicates Mexican ancestry in Los Angeles, California; YRI indicates Yoruba in Ibadan, Nigeria.

**Figure S2.** Quantile-Quantile plot for a genome-wide association study (GWAS) on the antidepressant effects to ketamine.

**Figure S3.** Manhattan plot for a genome-wide association study (GWAS) of genetic variation on the antidepressant effects to ketamine.

**Figure S4.** Regional association plot of rs55945116 on chromosome 15 containing the top signal in the genome-wide association study (GWAS) on the antidepressant effects to ketamine. The −log10 *p*-values for the single nucleotide polymorphisms (SNPs) are shown. SNPs are colored based on their *r^2^* with the labeled hit SNP (rs55945116), which has the smallest *p*-value in the region. Blue lines are estimates of recombination rates (cM/Mb) (right Y axis). Red, orange, green, and blue colors indicate the *r^2^* (derived from 1000 Genomes CEU data) between each plotted SNP and the top SNP (i.e. rs55945116, in purple).

**Figure S5.**  Regional association plot of rs112647602 on chromosome 6 containing the top signal in the genome-wide association study (GWAS) on the dissociation effects to ketamine. The −log10 *p*-values for the single nucleotide polymorphisms (SNPs) are shown. SNPs are colored based on their *r^2^* with the labeled hit SNP (rs112647602), which has the smallest *p*-value in the region. Blue lines are estimates of recombination rates (cM/Mb) (right Y axis). Red, orange, green, and blue colors indicate the *r^2^* (derived from 1000 Genomes CEU data) between each plotted SNP and the top SNP (i.e. rs112647602, in purple).

**Figure S6.** Quantile-Quantile plot for a genome-wide association study (GWAS) on the dissociation effects to ketamine.

**Figure S7.** Manhattan plot for a genome-wide association study (GWAS) of genetic variation on the dissociation effects to ketamine.

**Figure S8.** Regional association plot of rs17211233 on chromosome 5 containing the top signal in the genome-wide association study (GWAS) on the dissociation effects to ketamine. The −log10 *p*-values for the single nucleotide polymorphisms (SNPs) are shown. SNPs are colored based on their *r^2^* with the labeled hit SNP (rs17211233), which has the smallest *p*-value in the region. Blue lines are estimates of recombination rates (cM/Mb) (right Y axis). Red, orange, green, and blue colors indicate the *r^2^* (derived from 1000 Genomes CEU data) between each plotted SNP and the top SNP (i.e. rs17211233, in purple).

Table S1. Genomic regions with $p<1\times{10}^{-5}$ in the genome-wide association study (GWAS) on antidepressant effects to ketamine.

| CHR | SNP | BP | A1 | A2 | FRQ | INFO | BETA | SE | P | Gene (Distance) | Protein (Distance) |
| --- | --- | --- | --- | --- | --- | --- | --- | --- | --- | --- | --- |
| 15 | rs55945116 | 85220113 | G | C | 0.7892 | 0.9432 | 23.3347 | 4.4624 | 5.93E-07 | SEC11A (0) | SEC11A (0) |
| 15 | rs56385235 | 85329846 | C | T | 0.793 | 0.9483 | 23.1491 | 4.4595 | 7.05E-07 | ZNF592 (0) | ZNF592 (0) |
| 15 | rs11854597 | 85226810 | G | A | 0.7923 | 0.9652 | 22.689 | 4.4306 | 9.65E-07 | SEC11A (0) | SEC11A (0) |
| 15 | rs12917049 | 85238276 | G | A | 0.7925 | 0.9664 | 22.6703 | 4.4292 | 9.77E-07 | SEC11A (0) | SEC11A (0) |
| 15 | rs111783599 | 85237518 | G | A | 0.7924 | 0.9659 | 22.6656 | 4.4302 | 9.87E-07 | SEC11A (0) | SEC11A (0) |
| 15 | rs11073686 | 85284686 | A | T | 0.793 | 0.9697 | 22.5914 | 4.4281 | 1.05E-06 | ZNF592 (7180) | ZNF592 (7180) |
| 15 | rs8182086 | 85341859 | G | A | 0.7898 | 0.9641 | 22.2686 | 4.4187 | 1.39E-06 | ZNF592 (0) | ZNF592 (0) |
| 15 | rs8023889 | 85353603 | A | G | 0.7898 | 0.9641 | 22.2686 | 4.4187 | 1.39E-06 | ALPK3 (6308) | ALPK3 (6308) |
| 6 | rs112647602 | 54596755 | G | A | 0.7875 | 0.871 | -23.135 | 4.8666 | 4.82E-06 | KRASP1 (38626) | FAM83B (114814) |
| 4 | rs1846786 | 131919702 | T | G | 0.6951 | 0.8884 | -19.0893 | 4.0582 | 5.96E-06 |  |  |
| 22 | rs5997786 | 31254036 | C | T | 0.7995 | 0.8801 | 22.0315 | 4.6925 | 6.19E-06 | OSBP2 (0) | OSBP2 (0) |
| 2 | rs1524145 | 36122388 | G | T | 0.5 | 0.9682 | -17.3121 | 3.6948 | 6.44E-06 | MRPL50P1 (172180) | CRIM1 (460681) |
| 1 | rs6689906 | 116683540 | C | T | 0.9364 | 0.9278 | 35.6552 | 7.6306 | 6.80E-06 |  | MAB21L3 (29164) |
| 14 | rs28396435 | 38262484 | T | C | 0.9464 | 0.8565 | 40.2547 | 8.6242 | 6.94E-06 | TTC6 (0) | TTC6 (0) |
| 1 | rs11808840 | 116686398 | A | G | 0.9367 | 0.9124 | 35.9716 | 7.7281 | 7.34E-06 |  | MAB21L3 (32022) |
| 4 | rs12647096 | 131920334 | G | T | 0.6911 | 0.911 | -18.5248 | 3.9972 | 7.99E-06 |  |  |
| 4 | rs1500518 | 131923256 | T | G | 0.6911 | 0.911 | -18.5248 | 3.9972 | 7.99E-06 |  |  |
| 4 | rs13140684 | 131929376 | C | A | 0.6911 | 0.911 | -18.5248 | 3.9972 | 7.99E-06 |  |  |
| 4 | rs34591196 | 131932224 | G | A | 0.6911 | 0.911 | -18.5248 | 3.9972 | 7.99E-06 |  |  |
| 4 | rs28763721 | 131933159 | A | G | 0.6911 | 0.911 | -18.5248 | 3.9972 | 7.99E-06 |  |  |
| 4 | rs7653940 | 131939003 | G | A | 0.6911 | 0.911 | -18.5248 | 3.9972 | 7.99E-06 |  |  |
| 4 | rs12642294 | 131939529 | G | A | 0.6911 | 0.911 | -18.5248 | 3.9972 | 7.99E-06 |  |  |
| 4 | rs34869666 | 131940980 | C | A | 0.6914 | 0.91 | -18.5291 | 4.0002 | 8.07E-06 |  |  |
| 1 | rs79749176 | 116461018 | T | C | 0.9468 | 0.9306 | 39.0894 | 8.4686 | 8.63E-06 |  | SLC22A15 (58101) |
| 1 | rs1418662 | 116417733 | A | G | 0.9458 | 0.9414 | 38.3232 | 8.3266 | 9.13E-06 | HNRNPA1P43 (17293) | NHLH2 (31195) |
| 1 | rs7520783 | 116689587 | G | C | 0.937 | 0.9053 | 35.8988 | 7.8015 | 9.17E-06 |  | MAB21L3 (35211) |
| 1 | rs2797187 | 116432607 | T | C | 0.9459 | 0.9428 | 38.2787 | 8.3273 | 9.35E-06 |  | NHLH2 (46069) |
| 1 | rs113607929 | 116432803 | A | G | 0.9459 | 0.9428 | 38.2787 | 8.3273 | 9.35E-06 |  | NHLH2 (46265) |
| 4 | rs72662904 | 85312968 | A | G | 0.9389 | 0.9614 | 34.333 | 7.49 | 9.87E-06 |  | NKX6-1 (106635) |
| 1 | rs1689087 | 116539611 | C | A | 0.0514 | 0.9379 | -39.5839 | 8.6367 | 9.89E-06 | SLC22A15 (0) | SLC22A15 (0) |
| 18 | rs75908125 | 5155552 | G | C | 0.864 | 1.0926 | 22.8451 | 4.9849 | 9.91E-06 | C18orf42 (0) | C18orf42 (0) |

Chr=chromosome; SNP=single nucleotide polymorphism; A1=reference allele; A2=alternative allele; FRQ=frequency of A1 allele in all samples; INFO= information score of association; P=*p*-values of GWAS; Gene (Distance)= Gene name and distance to the start site of nearest gene the SNP is located within; Protein (Distance) = Protein name and distance to nearest protein coding gene start site.

Table S2. Genomic Regions with $p<1\times{10}^{-5}$ in the genome-wide association study (GWAS) on dissociation effects to ketamine.

| CHR | SNP | BP | A1 | A2 | FRQ | INFO | BETA | SE | P | Gene (Distance) | Protein (Distance) |
| --- | --- | --- | --- | --- | --- | --- | --- | --- | --- | --- | --- |
| 5 | rs17211233 | 80368763 | T | C | 0.9389 | 0.9349 | -26.9757 | 4.7058 | 1.90E-07 | RASGRF2 (0) | RASGRF2 (0) |
| 5 | rs72769298 | 80371244 | A | C | 0.9389 | 0.9349 | -26.9757 | 4.7058 | 1.90E-07 | RASGRF2 (0) | RASGRF2 (0) |
| 5 | rs72769300 | 80375872 | A | C | 0.9389 | 0.9349 | -26.9757 | 4.7058 | 1.90E-07 | RASGRF2 (0) | RASGRF2 (0) |
| 5 | rs72769297 | 80370541 | T | G | 0.9386 | 0.939 | -26.7584 | 4.6964 | 2.19E-07 | RASGRF2 (0) | RASGRF2 (0) |
| 3 | rs1400237 | 76172232 | T | G | 0.0778 | 1.0706 | 21.4345 | 4.165 | 2.02E-06 | ROBO2 (0) | ROBO2 (0) |
| 9 | rs12236015 | 122817820 | C | T | 0.833 | 1.1168 | -13.9838 | 2.7763 | 3.10E-06 |  |  |
| 9 | rs17407928 | 122807602 | C | T | 0.8333 | 1.114 | -14.004 | 2.7809 | 3.12E-06 |  | CDK5RAP2 (534846) |
| 9 | rs10984816 | 122811088 | G | A | 0.8333 | 1.1196 | -13.9691 | 2.7744 | 3.13E-06 |  | CDK5RAP2 (531360) |
| 9 | rs112829643 | 122811300 | C | T | 0.8333 | 1.1196 | -13.9691 | 2.7744 | 3.13E-06 |  | CDK5RAP2 (531148) |
| 9 | rs7865756 | 122814965 | C | T | 0.8333 | 1.12 | -13.9666 | 2.7744 | 3.14E-06 |  | CDK5RAP2 (527483) |
| 3 | rs6784961 | 76176756 | T | C | 0.082 | 1.0323 | 20.7645 | 4.1309 | 3.23E-06 | ROBO2 (0) | ROBO2 (0) |
| 9 | rs55689508 | 122842219 | G | A | 0.8301 | 1.0811 | -14.1485 | 2.8168 | 3.28E-06 |  | CDK5RAP2 (500229) |
| 9 | rs4407960 | 122825627 | C | T | 0.829 | 1.0948 | -13.95 | 2.79 | 3.58E-06 |  | CDK5RAP2 (516821) |
| 9 | rs12238332 | 122807099 | C | A | 0.8344 | 1.0971 | -14.0559 | 2.8144 | 3.67E-06 |  | CDK5RAP2 (535349) |
| 5 | rs17277296 | 80377287 | G | A | 0.926 | 1.0637 | -20.6883 | 4.1564 | 3.91E-06 | RASGRF2 (0) | RASGRF2 (0) |
| 9 | rs10818425 | 122834934 | T | A | 0.8272 | 1.0968 | -13.8268 | 2.7869 | 4.17E-06 |  | CDK5RAP2 (507514) |
| 5 | rs138480329 | 80392982 | G | C | 0.9209 | 1.0523 | -20.0933 | 4.0588 | 4.34E-06 | RASGRF2 (0) | RASGRF2 (0) |
| 3 | rs9713737 | 76297174 | A | G | 0.244 | 0.9868 | 13.5351 | 2.7481 | 4.79E-06 | ROBO2 (0) | ROBO2 (0) |
| 3 | rs17013853 | 76178350 | T | C | 0.9249 | 1.1234 | -20.1618 | 4.0963 | 4.86E-06 | ROBO2 (0) | ROBO2 (0) |
| 9 | rs12237217 | 122832381 | G | A | 0.8286 | 1.0832 | -13.8664 | 2.8176 | 4.87E-06 |  | CDK5RAP2 (510067) |
| 2 | rs77987715 | 65955079 | A | G | 0.6547 | 1.0199 | -11.7152 | 2.3832 | 4.97E-06 |  | SPRED2 (295308) |
| 5 | rs145655910 | 80395795 | C | T | 0.9167 | 1.0547 | -19.4753 | 3.9816 | 5.47E-06 | RASGRF2 (0) | RASGRF2 (0) |
| 4 | rs3900502 | 189869233 | C | A | 0.4877 | 0.8444 | -12.2832 | 2.5129 | 5.53E-06 |  | TRIML1 (808660) |
| 11 | rs3018154 | 94420308 | C | T | 0.9111 | 1.0392 | -19.9461 | 4.0971 | 5.97E-06 |  | AMOTL1 (19289) |
| 11 | rs2851574 | 94420322 | C | T | 0.9111 | 1.0392 | -19.9461 | 4.0971 | 5.97E-06 |  | AMOTL1 (19275) |
| 11 | rs598480 | 94421141 | A | G | 0.9111 | 1.0392 | -19.9461 | 4.0971 | 5.97E-06 |  | AMOTL1 (18456) |
| 11 | rs11020906 | 94425118 | G | A | 0.0889 | 1.0396 | 19.9406 | 4.097 | 6.00E-06 |  | AMOTL1 (14479) |
| 11 | rs11020907 | 94425284 | G | A | 0.0889 | 1.0396 | 19.9406 | 4.097 | 6.00E-06 |  | AMOTL1 (14313) |
| 11 | rs11020909 | 94426502 | T | C | 0.0889 | 1.0396 | 19.9392 | 4.097 | 6.01E-06 |  | AMOTL1 (13095) |
| 11 | rs10430825 | 94427573 | T | C | 0.0889 | 1.0396 | 19.9392 | 4.097 | 6.01E-06 |  | AMOTL1 (12024) |
| 11 | rs10741495 | 94447525 | C | T | 0.0889 | 1.0396 | 19.9392 | 4.097 | 6.01E-06 | AMOTL1 (0) | AMOTL1 (0) |
| 11 | rs4269878 | 94453793 | T | C | 0.0889 | 1.0396 | 19.9392 | 4.097 | 6.01E-06 | AMOTL1 (0) | AMOTL1 (0) |
| 11 | rs4475878 | 94453903 | A | G | 0.0889 | 1.0396 | 19.9392 | 4.097 | 6.01E-06 | AMOTL1 (0) | AMOTL1 (0) |
| 11 | rs7104535 | 94454413 | A | G | 0.0889 | 1.0396 | 19.9392 | 4.097 | 6.01E-06 | AMOTL1 (0) | AMOTL1 (0) |
| 11 | rs1815907 | 94455919 | G | A | 0.0889 | 1.0388 | 19.927 | 4.0977 | 6.10E-06 | AMOTL1 (0) | AMOTL1 (0) |
| 20 | rs1287071 | 5828375 | A | G | 0.7111 | 1.1346 | -11.3492 | 2.3349 | 6.15E-06 | C20orf196 (0) | C20orf196 (0) |
| 2 | rs11127199 | 29213561 | G | A | 0.6335 | 0.995 | 11.445 | 2.3621 | 6.53E-06 | FAM179A (0) | FAM179A (0) |
| 2 | rs6547901 | 29214258 | A | G | 0.6357 | 0.9762 | 11.5657 | 2.3952 | 6.95E-06 | FAM179A (0) | FAM179A (0) |
| 2 | rs6547902 | 29214350 | T | A | 0.6361 | 0.9739 | 11.5789 | 2.3998 | 7.06E-06 | FAM179A (0) | FAM179A (0) |
| 11 | rs2510950 | 94414398 | C | G | 0.8968 | 0.9813 | -18.9919 | 3.9545 | 7.69E-06 |  | AMOTL1 (25199) |
| 2 | rs7578007 | 29215005 | G | T | 0.6444 | 0.9332 | 11.9236 | 2.4829 | 7.70E-06 | FAM179A (0) | FAM179A (0) |
| 20 | rs6053740 | 5827061 | G | A | 0.708 | 1.1167 | -11.2709 | 2.3487 | 7.80E-06 | C20orf196 (0) | C20orf196 (0) |
| 3 | rs4855976 | 76267368 | A | G | 0.0702 | 0.7747 | 23.7477 | 4.9636 | 8.24E-06 | ROBO2 (0) | ROBO2 (0) |
| 3 | rs6549848 | 76266426 | T | C | 0.0702 | 0.7754 | 23.7226 | 4.9597 | 8.28E-06 | ROBO2 (0) | ROBO2 (0) |
| 20 | rs11907319 | 42923299 | T | C | 0.8099 | 0.9753 | 14.3351 | 3.0037 | 8.63E-06 | FITM2 (16510) | FITM2 (16510) |
| 20 | rs1287021 | 5824893 | T | C | 0.7068 | 1.1139 | -11.2057 | 2.3508 | 8.82E-06 | C20orf196 (0) | C20orf196 (0) |
| 11 | rs3016624 | 94415856 | A | C | 0.8956 | 0.9741 | -18.8758 | 3.9639 | 8.98E-06 |  | AMOTL1 (23741) |
| 9 | rs67317315 | 122814339 | T | C | 0.8167 | 1.0724 | -13.346 | 2.8036 | 9.03E-06 |  | CDK5RAP2 (528109) |
| 20 | rs1287022 | 5824007 | C | T | 0.7065 | 1.1138 | -11.181 | 2.3511 | 9.20E-06 | C20orf196 (0) | C20orf196 (0) |
| 20 | rs1287023 | 5823795 | G | A | 0.7064 | 1.1136 | -11.1776 | 2.3511 | 9.25E-06 | C20orf196 (0) | C20orf196 (0) |
| 20 | rs1287024 | 5823642 | C | T | 0.7064 | 1.1137 | -11.1718 | 2.3511 | 9.33E-06 | C20orf196 (0) | C20orf196 (0) |
| 15 | rs4558394 | 98322385 | A | G | 0.85 | 0.9108 | -15.6495 | 3.2958 | 9.46E-06 | LINC00923 (0) | LINC00923 (0) |

Chr=chromosome; SNP=single nucleotide polymorphism; A1=reference allele; A2=alternative allele; FRQ=frequency of A1 allele in all samples; INFO= information score of association; P=*p*-values of GWAS; Gene (Distance)= Gene name and distance to the start site of nearest gene the SNP is located within; Protein (Distance) = Protein name and distance to nearest protein coding gene start site.

Table S3. Polygenic risk score analysis.

|  |  |  | LD r2 < 0.5 | |  |  | LD r2 < 0.2 | |
| --- | --- | --- | --- | --- | --- | --- | --- | --- |
| Scopolamine | Threshold* | R^2^ | Nsnps | *p*-value |  | R^2^ | Nsnps | *p*-value |
|  | *p*<0.01 | 0.0014 | 3950 | 0.844 |  | 0.0121 | 1225 | 0.560 |
|  | *p*<0.05 | 0.0010 | 19731 | 0.869 |  | 0.0002 | 6557 | 0.944 |
| CEU | *p*<0.1 | 0.0042 | 39812 | 0.732 |  | 0.0017 | 13663 | 0.825 |
| (N=37) | *p*<0.2 | 0.0370 | 82679 | 0.305 |  | 0.0286 | 29850 | 0.368 |
|  | *p*<0.3 | 0.0279 | 129643 | 0.374 |  | 0.0581 | 48474 | 0.196 |
|  | *p*<0.4 | 0.0364 | 183036 | 0.309 |  | 0.0461 | 70012 | 0.251 |
|  | *p*<0.5 | 0.0349 | 250828 | 0.319 |  | 0.0602 | 97129 | 0.187 |
|  | *p*<0.01 | 0.0017 | 3950 | 0.744 |  | 0.0189 | 1225 | 0.277 |
|  | *p*<0.05 | 0.0056 | 19731 | 0.557 |  | 0.0212 | 6557 | 0.250 |
| ALL | *p*<0.1 | 0.0143 | 39812 | 0.346 |  | 0.0222 | 13663 | 0.238 |
| (N=69) | *p*<0.2 | 0.0588 | 82679 | 0.052 |  | 0.0793 | 29850 | 0.023 |
|  | *p*<0.3 | 0.0445 | 129643 | 0.093 |  | 0.0522 | 48474 | 0.068 |
|  | *p*<0.4 | 0.0628 | 183036 | 0.045 |  | 0.0769 | 70012 | 0.026 |
|  | *p*<0.5 | 0.0648 | 250828 | 0.041 |  | 0.1095 | 97129 | 0.007 |

**p* is the predetermined significance of the discovery sample, drawn from the ketamine data comprising 157 subjects of European ancestry. The overlapped SNPs were pruned based on linkage disequilibrium (LD) (*r*^2^ < 0.5 and *r*^2^ < 0.2). The target sample is drawn from the scopolamine data, comprising 37 subjects of European ancestry or all 69 subjects.
