# Supplementary figures and images for: Exploratory genome-wide association analysis of response to ketamine and a polygenic analysis of response to scopolamine in depression
Source: Transl Psychiatry. 2018 Dec 14;8:280. doi: 10.1038/s41398-018-0311-7 (PMC6294748; doi:10.1038/s41398-018-0311-7)

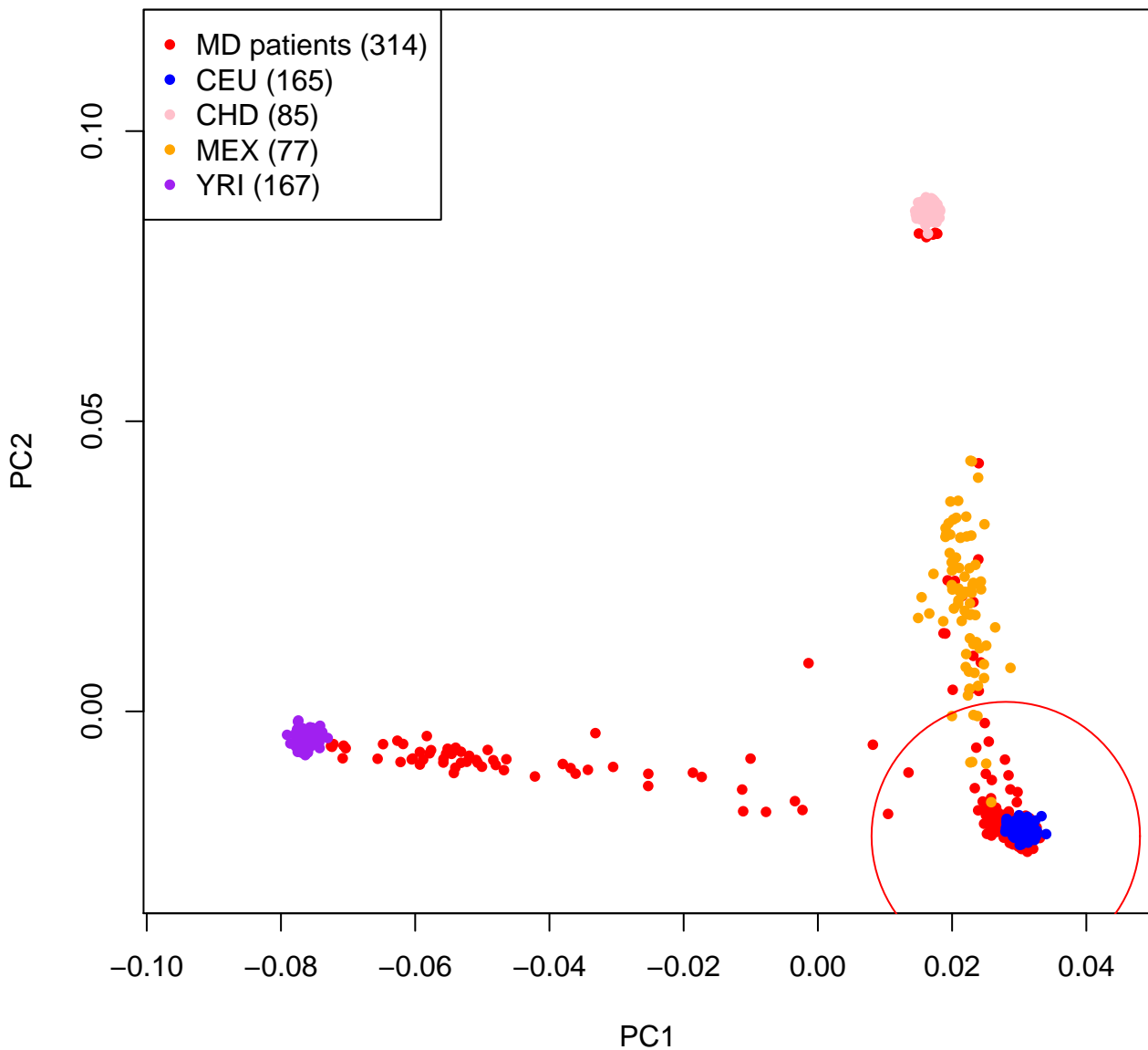

Supplement: Supplementary file 2 — Figure S1 [file 41398_2018_311_MOESM2_ESM.pdf]

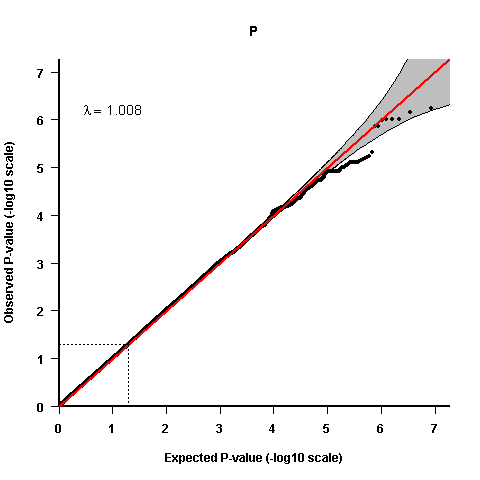

Supplement: Supplementary file 3 — Figure S2 [file 41398_2018_311_MOESM3_ESM.png]

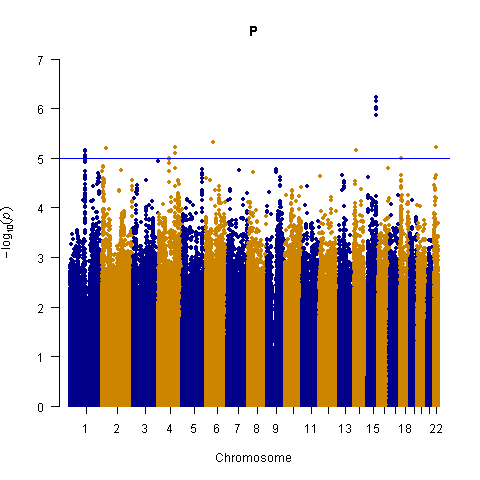

Supplement: Supplementary file 4 — Figure S3 [file 41398_2018_311_MOESM4_ESM.png]

Plotted SNPs

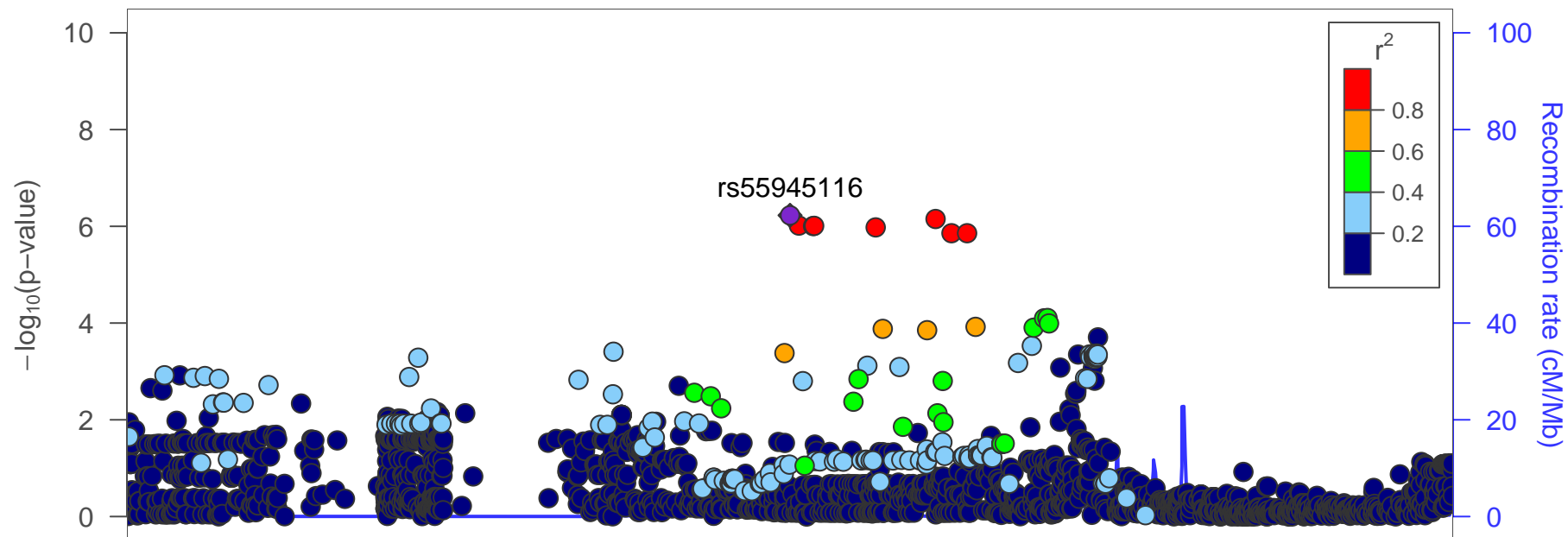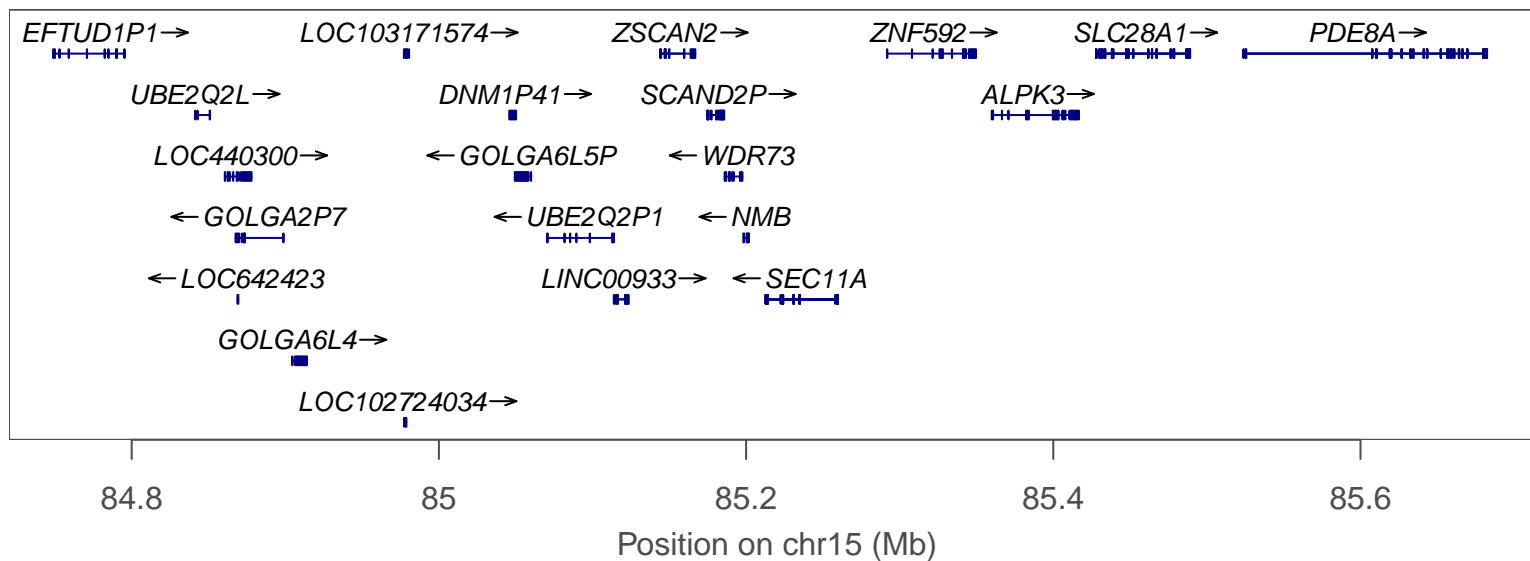

Supplement: Supplementary file 5 — Figure S4 [file 41398_2018_311_MOESM5_ESM.pdf]

Plotted SNPs

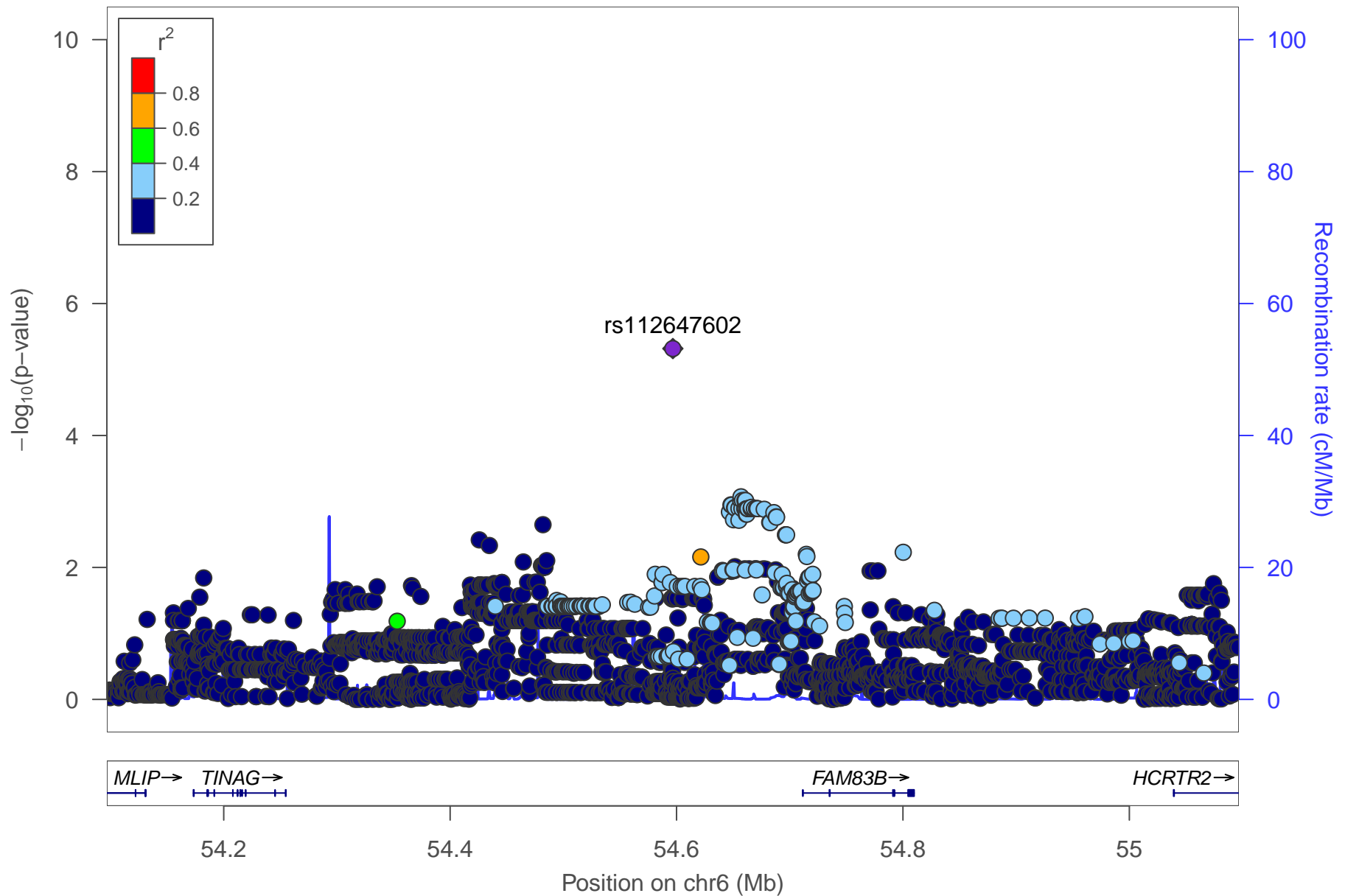

Supplement: Supplementary file 6 — Figure S5 [file 41398_2018_311_MOESM6_ESM.pdf]

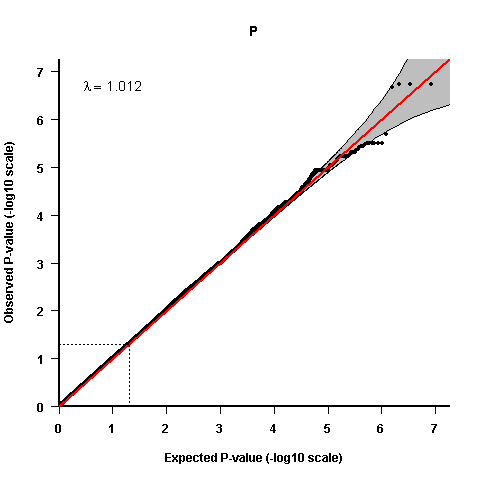

Supplement: Supplementary file 7 — Figure S6 [file 41398_2018_311_MOESM7_ESM.png]

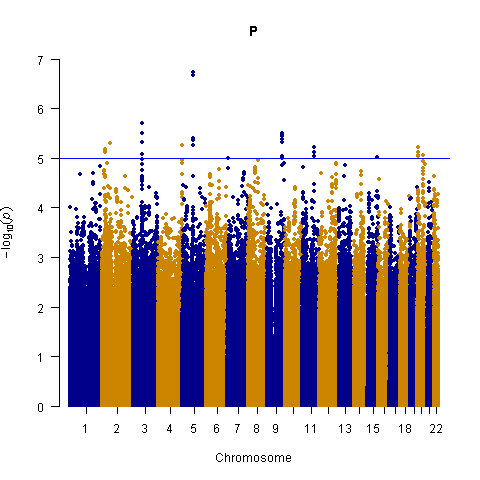

Supplement: Supplementary file 8 — Figure S7 [file 41398_2018_311_MOESM8_ESM.png]

Plotted SNPs

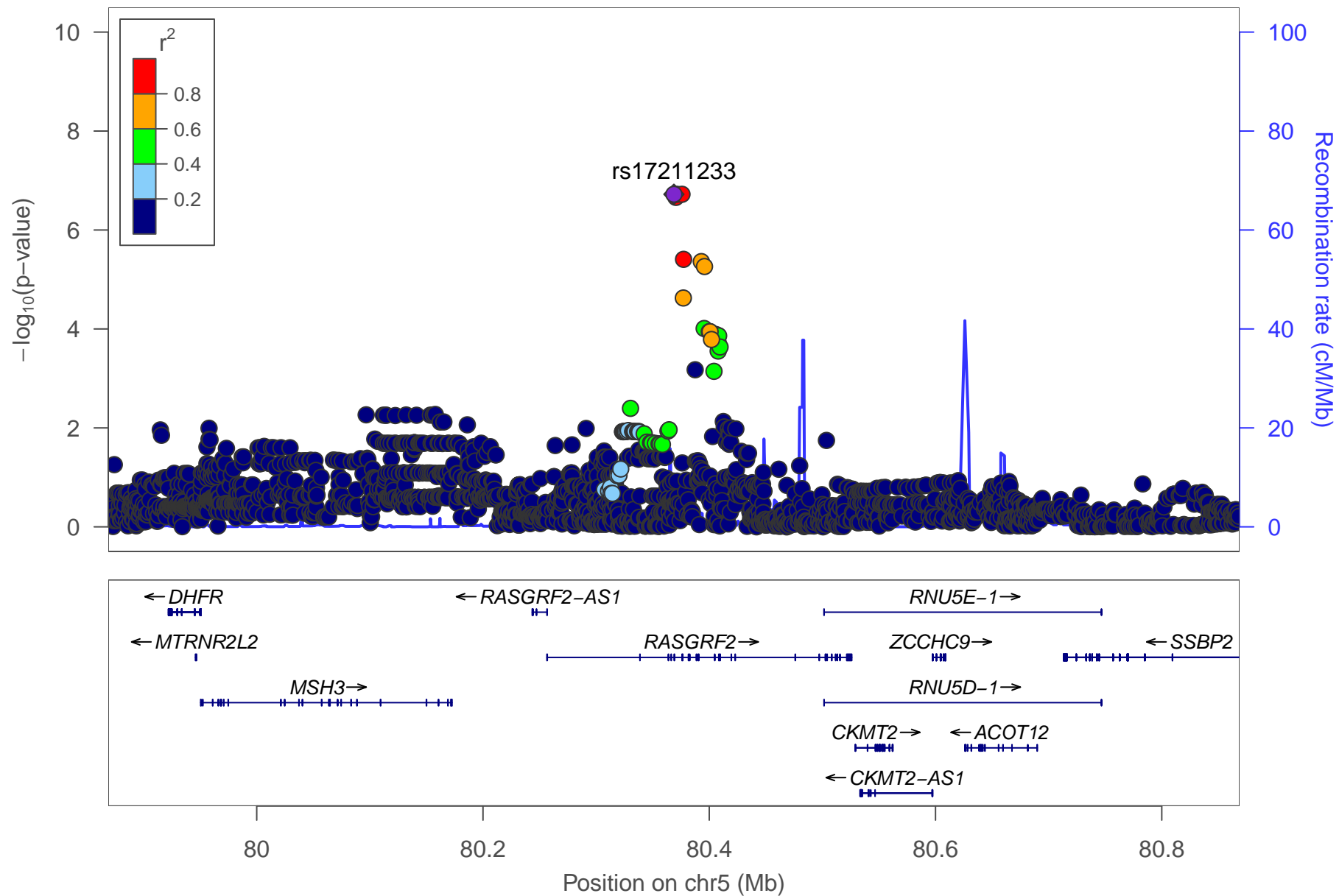

Supplement: Supplementary file 9 — Figure S8 [file 41398_2018_311_MOESM9_ESM.pdf]
